# Tumor Regression and Patterns of Distant Metastasis of T1-T2 Nasopharyngeal Carcinoma with Intensity-Modulated Radiotherapy

**DOI:** 10.1371/journal.pone.0154501

**Published:** 2016-04-27

**Authors:** Ming-Yao Wu, Xia-Yun He, Chao-Su Hu

**Affiliations:** 1 Department of Radiation Oncology, Fudan University Shanghai Cancer Center, Shanghai, China; 2 Department of Oncology, Shanghai Medical College, Shanghai, China; University of Nebraska Medical Center, UNITED STATES

## Abstract

**Purpose:**

To study tumor regression and failure patterns in T1-T2 non-metastatic nasopharyngeal carcinoma (NPC) after intensity-modulated radiotherapy (IMRT).

**Methods:**

A retrospective analysis of 139 nasopharyngeal carcinoma patients treated with IMRT between January 2005 and December 2010 in our center was performed. According to the AJCC staging system, all primary lesions were attributed to T1 and T2. The prescription doses were 66 Gy at 30 fractions to gross tumor volume of the nasopharynx and the positive neck nodes, 60 Gy to high-risk clinical target volume and 54 Gy to low-risk clinical target volume. Patients staged III, IV A/B or II (lymph node measured 4 cm or more in diameter) received platinum-based chemotherapy.

**Results:**

By the end of radiotherapy, 7.2% (10/139), 23.7% (33/139), and 9.4% (13/139) of patients had residual lesions in the nasopharynx, cervical lymph nodes and retropharyngeal lymph nodes, respectively. The majority of patients had complete remission within 6 months of radiotherapy completion. Five months after IMRT, three patients with residual tumors in the cervical lymph nodes underwent surgery. Among these patients, two patients had positive pathological findings, and one patient had negative findings. With a median follow-up of 59 months, the 5-year overall survival, local control, regional control and distant metastasis-free rates were 87.8%, 96.7%, 94.9% and 89.1%, respectively. Fifteen patients developed distant metastases, representing the primary failure pattern.

**Conclusions:**

Most residual lesions that persisted after IMRT vanished completely in six months. Considering the potential damage to normal structures, clinicians should be cautious when considering the use of boost irradiation after radiotherapy. Distant metastasis was the primary cause of treatment failure, which was significantly higher in N2-3 patients than in N0-1. Additional studies to better understand distant metastases are needed.

## Introduction

Nasopharyngeal carcinoma (NPC) is a common malignancy in southern China and is highly sensitive to radiotherapy. With the technical superiority of intensity-modulated radiotherapy (IMRT), clinical outcomes such as loco-regional control have been significantly enhanced. Compared to 2-dimensional radiotherapy (2DRT), IMRT can deliver a higher dose of radiation to the tumor while sparing normal tissues. Previous studies have shown that IMRT improves disease control and reduces adverse effects, especially in early T-stage disease [1–3]. Based on 2DRT, several randomized controlled trials [4–7] and meta-analysis [8] have confirmed concurrent chemoradiotherapy as the standard treatment for loco-regionally advanced NPC. However, the value of concurrent chemotherapy in NPC treated with IMRT is controversial. A recent study found that no benefit was achieved in local control or survival by concurrent chemotherapy for patients with stage III/IVa-b disease treated with IMRT [9].

Moreover, IMRT and conventional radiotherapy possess different radiobiological effects. It is very difficult to choose a suitable time point for evaluation of a residual tumor, as all primary lesions after IMRT have received a radical radiation dose. Furthermore, it is not clear if all residual lesions need to receive boost irradiation. Although excellent locoregional control can be achieved with IMRT for NPC, the distant metastasis rates are still high, representing a predominant failure pattern [10–12]. Therefore, in this study, we retrospectively analyzed 5-year treatment results for patients with T1-2 NPC treated with IMRT to observe tumor regression and treatment failures in IMRT used to treat early T-stage NPC patients.

## Materials and Methods

### Patients and pretreatment evaluation

Between January 2005 and December 2010, a total of 139 T1-T2 patients with histologically diagnosed non-metastatic NPC treated by definitive IMRT were enrolled in this study. All patients were histologically proven to have World Health Organization (WHO) type II/III NPC. Pretreatment evaluations consisted of a medical history and physical examination, blood chemistry tests, chest X-ray/computed tomography (CT), abdominal ultrasound/CT, enhanced magnetic resonance imaging (MRI) of the nasopharynx and neck, nasopharyngoscopy and bone emission computed tomography (ECT). Additional tests were offered for those with suspicious findings. Dental extraction, if deemed necessary, was performed before radiation therapy. All patients underwent disease staging according to the AJCC staging system. The study was conducted according to the principles expressed in the Declaration of Helsinki and approved by the institutional review board of Fudan University Shanghai Cancer Center. All patients provided informed written consent to participate in this study.

### Radiotherapy

Patients were immobilized in the supine position with thermoplastic masks. CT planning scans using 5 mm slices with intravenous contrast material were performed. An 88-cm aperture CT (Philips) was used for analog positioning, and the CT images were transferred to the treatment planning system through LAN.

Gross tumor volume (GTV) included the primary tumor and metastatic lymph nodes found in clinical and imaging examination. The clinical target volume (CTV) included the nasopharynx, retropharyngeal lymph node, skull base, anterior one-third of the clivus, pterygoid fossa, parapharyngeal space, inferior sphenoid sinus, posterior one-third of the nasal cavity and maxillary sinus, and drainage of the upper neck (levels II, III and Va in N0 patients and levels IV-Vb in N1-N3 patients). Critical normal structures, including the brainstem, spinal cord, optic nerves, chiasm, lens, eyeballs, temporal lobes and parotid glands, were carefully delineated.

A total dose of 66 Gy/30 F was prescribed to the planning target volume (PTVg), defined as the GTV, with a 0.5 cm margin. PTV60 (high-risk clinical target volume) covering the CTV and a 0.5 cm margin was prescribed 60 Gy/30 F. PTV54 (low-risk clinical target volume) was prescribed 54 Gy/30 F. All patients were treated with one fraction daily, for five days per week. Treatment was delivered with an Elekta Precise or Synergy Linear Accelerator. An inverse planning software (ADAC Pinnacle 7.4 or 7.6) was used for plan optimization.

### Chemotherapy

All patients staged III, IV A/B or II (lymph node measured 4 cm or more in diameter) received platinum-based chemotherapy. A total of 106 (76.3%) patients received chemotherapy, including neoadjuvant chemotherapy, concurrent chemotherapy and adjuvant chemotherapy. Concurrent and non-concurrent (neoadjuvant ± adjuvant) chemotherapy was given to 20 and 86 patients, respectively. A total of 78 patients received adjuvant chemotherapy. Neoadjuvant chemotherapy was given every 3 weeks. Four weeks after the completion of RT, adjuvant chemotherapy was administered every 3 weeks. The common regimens of neoadjuvant and adjuvant chemotherapy included 2 cycles of GP (gemcitabine 1000 mg/m^2^/day, day 1, day 8, and cisplatin 25 mg/m^2^/day, days 1–3), TPF (docetaxel 60 mg/m^2^/day, day 1, cisplatin 25 mg/m^2^/day, days 1–3, and 5-fluorouracil 0.5 g/m^2^/day, days 1–5) and PF (cisplatin 25 mg/m^2^/day, days 1–3, and 5-fluorouracil 0.5 g/m^2^/day, days 1–5). Concurrent chemotherapy mainly consisted of cisplatin 30–40 mg/m^2^ weekly during radiation.

### Assessment and follow-up

Patients were evaluated weekly during radiation therapy. After treatment completion, follow-ups occurred every 3 months for the first 2 years, every 6 months from the second through the fifth year and annually thereafter. Each follow-up included medical history, physical examination and nasopharyngoscopy. Enhanced MRI of the nasopharynx and neck areas was performed every 6 to 12 months after treatment. Chest X-ray and ultrasonography of the abdomen were conducted once yearly. Additional tests were ordered whenever there was any clinical indication.

### Statistics

The times to local/regional failure and distant metastases were calculated from the start of treatment to the dates of recurrence and metastases, respectively. Statistical Package for Social Sciences (SPSS version 20.0) software was used for statistical analyses. The Kaplan-Meier method was used to calculate local control (LC), distant metastasis-free (DMF) and overall survival (OS) rates. The log-rank test was used to assess the significance of differences in DMF and OS.

## Results

### Patient characteristics

Of the 139 consecutive NPC patients with definite pathological diagnosis (WHO II-III), 106 were males and 33 were females. The median age was 49 years (range, 18–73 years). The patient characteristics are shown in Table 1. The distributions of the T and N classification as well as the clinical stages (AJCC) are listed in Table 1. Before diagnosis, 28.8% and 10.1% of patients underwent fine needle aspiration cytology and neck mass biopsy, respectively.

**Table 1:** Patient characteristics.

Characteristics	Patients(%)
Gender	
Male	106(76.3)
Female	33(23.7)
Age	
<50	75(54.0)
≥50	64(46.0)
TNM Stage	
T1	52(37.4%)
T2	87(62.6%)
N0	23(16.5%)
N1	48(34.5%)
N2	38(27.3%)
N3	30(21.6%)
AJCC stage	
Stage I	16(11.5%)
Stage II	55(39.6%)
Stage III	38(27.3%)
Stage IV	30(21.6%)

### Tumor regression and treatment outcomes

All patients received IMRT. The median total dose of GTV was 66 Gy (range, 59.4–70.4 Gy). The median duration was 43 days (range, 37–71 days), and the median number of fractions was 30 (range, 27–32 fractions).

By the end of radiotherapy, 7.2% (10/139) of patients had residual nasopharyngeal lesions as determined by MRI and nasopharyngoscopy. Four patients received boost doses (8 Gy in 3 cases, 7 Gy in 1 case) at the primary site by brachytherapy to the residual disease 2 to 3 weeks after external radiation, and three patients received 4.4 Gy/2 F by boost external irradiation just after the planned treatment. Eight patients with residual nasopharyngeal lesions had complete remission 3 months after radiotherapy, and two patients had complete remission after 5 months. Similarly, thirty-three patients had residual cervical lymph node disease (four patients received 4.4 Gy/2 F by boost external irradiation radiotherapy just after the planned treatment), 29 of whom had complete remission 3 months after radiotherapy and 1 case had complete remission after 5 months. Salvage surgery was performed in three patients with persistent lesions 5 months after the completion of radiotherapy; two were positive, and one was negative pathologically. Thirteen patients had residual retropharyngeal lymph node lesions (five patients received 4.4 Gy/2 F by boost external irradiation radiotherapy just after the planned treatment) and, of these, 10 and 3 patients had complete remission within 3 and 6 months after the completion of radiotherapy, respectively.

With a median follow-up of 59 months (range, 13–98 months), seven patients had recurrence in the nasopharynx. Of these, 2/52 were T1 and 5/58 were T2. Five cases relapsed 36 months after IMRT. The 5-year local control rates for patients with T1 and T2 diseases were 94.9 and 97.7%, respectively (P = 0.664). The 5-year local control rate for all patients was 96.7%.

Seven patients had regional recurrence from 5 to 27 months after IMRT, and 3 and 4 of these patients had N2 and N3 disease, respectively.

**Fig 1:**
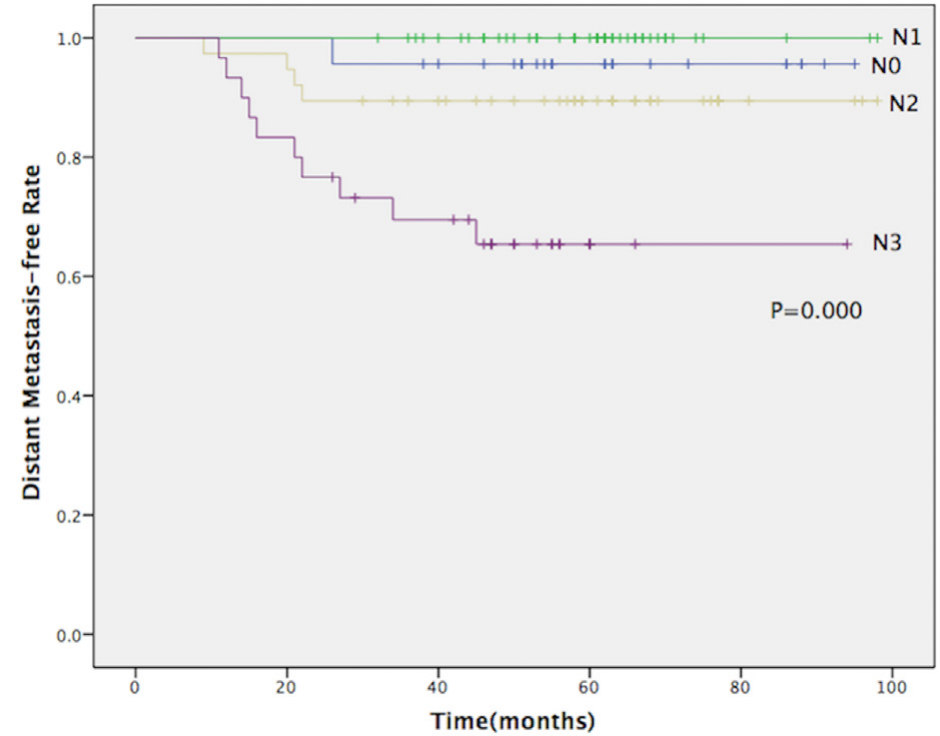
Distant metastasis-free rate for all patients according to N stage.

Fifteen patients developed distant metastasis. Of these, 1 had N0 disease, none had N1, 4 had N2 and 10 had N3 disease. Fourteen patients were found to have distant metastasis within 36 months after treatment. The Bone was the most frequent site for distant metastasis. The characteristics of patients developing distant failure are listed in Table 2. The 5-year DMF rates for patients with N0, N1, N2 and N3 diseases were 95.7%, 100.0%, 89.5% and 65.4%, respectively (P = 0.000)(Fig 1). The 5-year DMF rates for patients with T1 and T2 disease were 90.0% and 88.4%, respectively (P = 0.696).

**Table 2:** Details of patients developing distant metastases after treatment.

No.	Stage	Metastasis Time(months)	Site Of Metastasis	Status
1	T1N3	45	Bone	Alive
2	T1N0	26	Multiple 3	Dead
3	T1N3	15	Multiple 2	Alive
4	T1N3	21	Liver and lung	Dead*
5	T1N3	22	Liver and lung	Dead
6	T2N2	20	Bone	Dead*
7	T2N2	22	Multiple 1	Dead
8	T2N2	21	Multiple 1	Dead
9	T2N2	9	Multiple 1	Dead
10	T2N3	27	Lung	Alive
11	T2N3	12	Multiple 1	Dead
12	T2N3	34	Bone	Alive
13	T2N3	14	Liver	Dead
14	T2N3	16	Bone	Dead
15	T2N3	11	Multiple 1	Dead

*Died of distant metastasis accompanied by recurrence in the regional lymph nodes

Multiple 1: Bone+liver and/or lung; Multiple 2: Other sites with coexisting bone metastasis; Multiple 3: Other sites without coexisting bone metastasis.

### Second primary cancers

A total of 5 patients developed second primary cancer after IMRT. One patient had acinic cell carcinoma of the parotid 96 months after IMRT and three developed lung cancer 9, 31, and 73 months after radiotherapy as confirmed by pathology. The other patient died of melanoma of the foot, which had been treated by surgical excision, 12 months after IMRT.

### Survival

**Fig 2:**
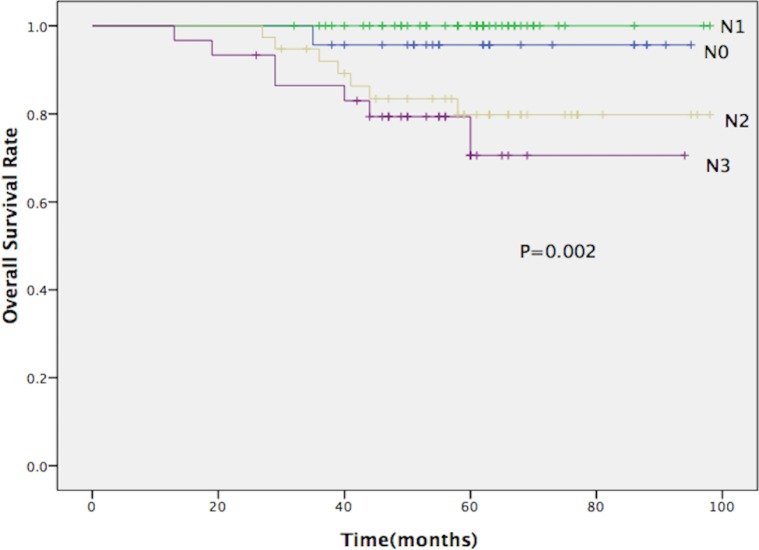
Overall survival rate for all patients according to N stage.

Fifteen patients died prior to the current analysis. The causes of death included the following: nine patients died of distant metastasis, two of distant metastasis accompanied by recurrence in the regional lymph nodes, one of retropharyngeal recurrence, one of secondary malignancy, one of non-neoplastic disease and one of unknown causes. The 5-year overall survival, local control, regional control and distant metastasis-free rates for all patients were 87.8%, 96.7%, 94.9% and 89.1%, respectively. The 5-year OS rates for patients with N0, N1, N2 and N3 diseases were 95.7, 100, 79.8 and 70.5%, respectively (P = 0.002; Fig 2).

## Discussion

For non-metastatic NPC patients, radiotherapy +/- chemotherapy is the primary treatment regimen. Because of the high radiation dose and need for adequate coverage of high-risk sites, IMRT has become the preferred technique instead of 2DRT and three-dimensional RT (3DRT). A study from Hong Kong reported by Lee et al. demonstrated the treatment outcomes for NPC patients with various techniques, including 2DRT, 3DRT and IMRT. The 5-year disease-specific survival (DSS) improved from 78% in the 2DRT era to 85% in the IMRT era, while the incidence of serious toxicities decreased [10].

IMRT can improve target volume coverage and increase the radiation dose to the gross tumor while sparing normal structures. Several studies have reported greater than 85% local control rates [3, 11, 13]. Su et al. reported the 5-year results of 198 early-stage (T1-T2bN0-N1M0) NPC patients treated with IMRT alone. The 5-year estimated disease-specific survival, local recurrence-free survival, and distant metastasis-free survival rates were 97.3%, 97.7%, and 97.8%, respectively [13].

As lymphatic drainage of the nasopharynx is predominantly to the cervical lymph nodes, the first disease sign in many patients is often the appearance of cervical lymphadenopathy. Several studies have shown that approximately 75.4%-86.4% patients have cervical lymph node metastases at diagnosis [14–16]. In the current study, patients in both the T1 and T2 stages had cervical lymph node involvement, although the volume of the primary tumor in these stages is relatively small. Some of the patients underwent fine needle aspiration cytology or neck mass biopsy to assist in the diagnosis. Often, the primary lesions in T1 and T2 patients are quite small and the nasopharyngeal cavity needs to be examined very carefully, correlating suspicious changes with the results of Epstein-Barr viral DNA analysis, magnetic resonance imaging (MRI) and/or positron emission tomography CT scan [17, 18].

It is still unclear if boost irradiation should be performed in T1-2 cases, especially when the dose to the gross tumor volume is considered sufficient. Many studies have found that a high proportion of residual disease disappear spontaneously at the end of radiotherapy [19, 20]. In a study of 188 patients, Zhang et al. observed that 40.4% of patients had CR, 44.7% had PR, and 14.9% had SD of the nasopharyngeal lesion at the end of radiotherapy; however, 3–4 months after RT, 97.4% of patients had CR and only 2.6% had persistent nasopharyngeal lesions [19]. Nasopharyngeal lesions appear to react differently in response to conventional radiotherapy versus IMRT. In the 1990s, Kwong et al. reported the results of 803 patients who underwent post-radiotherapy nasopharyngeal biopsies. In 16.3% of the patients, spontaneous remission was observed on repeat biopsies after initial positive histology, and patients with early and delayed histologic remission had 5-year NPC control rates of 82.4% and 76.8%, respectively (P = 0.35) [20]. However, the radiobiological effect of IMRT is somewhat different, and seems to require more time for tumor regression. In the present study, 10 patients had residual nasopharyngeal lesions, 33 had residual cervical lymph node lesions and 13 had residual retropharyngeal lymph node lesions. The majority had a complete response within 5 months after radiotherapy; therefore, clinicians should be cautious as to whether boost irradiation is necessary after radical radiotherapy. Xu et al. evaluated the value of concurrent chemotherapy in T1-2N1 NPC patients treated with IMRT. At the end of RT, the CR rates in the investigational and control arms were both 72.1%. Most of the patients received boost irradiation and 95.3% had CR 3 months after RT [21]. Therefore, it would seem that the need for concurrent chemotherapy is more dependent on the status of the cervical lymph nodes than on the extent of the primary tumor.

Sun et al. analyzed 868 NPC patients treated with IMRT and found that the majority of locoregional recurrence occurred within 5 years after treatment. Locoregional recurrence was quite rare (5.3%) after 5 years [9]. This study suggested that close follow-up for 5 years after treatment is necessary to rapidly detect recurrent lesions. Our data showed that seven patients developed local recurrence in the nasopharynx, and the majority (5/7, 71.4%) occurred after 36 months. The median local recurrence time was 52 (13–78) months. Due to the excellent efficacy and prolonged survival time, long-term follow-up is very essential and can help identify recurrences in time.

Even in the IMRT era, distant metastasis has become the major cause of death for NPC patients. The present study shows that 60% (9/15) of patients died due to distant metastasis. With the dosage advantage of IMRT, excellent loco-regional control for NPC was achieved, especially for T1-T2 cases. In the 2DRT era, some studies reported that the N stage was an important prognostic factor in NPC [22, 23]. Although IMRT has qualitative changes in treatment mode, many studies have reported that the N stage was still an independent prognostic factor affecting the distant metastasis-free and overall survival rates [15, 24–27]. A series from The Cancer Hospital of Fujian Medical University reported on 370 NPC patients treated with IMRT and found that the N classification was a significant prognostic factor for both metastasis-free survival and overall survival. The study showed that patients with stage N2-3 had a higher risk of developing distant metastasis (N2-N3 vs. N0-N1: HR = 5.792, P = 0.029, 95% CI: 1.279–3.098) [24]. In our study, a similar result was obtained. Patients staged III, IV A/B or II (lymph node measuring 4 cm or more in diameter) received radiotherapy combined with platinum-based chemotherapy. The 5-year DMF rate for all patients was 89.1%. Patients with an advanced N stage had worse outcomes. The 5-year DMF rates for patients with N0, N1, N2 and N3 diseases were 95.7%, 100.0%, 89.5% and 65.4%, respectively (P = 0.000). Distant metastasis has become the predominant failure pattern in NPC patients undergoing IMRT; thus, more potent systemic therapies are needed for patients with N2 and N3 diseases.

## Conclusion

Our study showed that IMRT provided excellent local control for T1-T2 NPC. Most residual tumors that were present after IMRT completely recede after 5 months. Therefore, if we take into consideration the damage to normal tissues, the use of boost irradiation to residual lesions should be used judiciously when the doses given to the primary lesion were deemed adequate. Distant metastasis was the main pattern of treatment failure. We found that N2-3 stage patients had higher risks of developing distant metastases. Further researches on systemic therapy for distant metastases are urgently needed.

## Supporting Information

S1 Data Relevant data underlying the findings described in manuscript. (XLS)
